# An *in vivo* genetic screen for genes involved in spliced leader *trans-*splicing indicates a crucial role for continuous *de novo* spliced leader RNP assembly

**DOI:** 10.1093/nar/gkx500

**Published:** 2017-06-05

**Authors:** Lucas Philippe, George C. Pandarakalam, Rotimi Fasimoye, Neale Harrison, Bernadette Connolly, Jonathan Pettitt, Berndt Müller

**Affiliations:** School of Medicine, Medical Sciences and Nutrition, University of Aberdeen, Institute of Medical Sciences, Foresterhill, Aberdeen AB25 2ZD, UK

## Abstract

Spliced leader (SL) *trans*-splicing is a critical element of gene expression in a number of eukaryotic groups. This process is arguably best understood in nematodes, where biochemical and molecular studies in *Caenorhabditis elegans* and *Ascaris suum* have identified key steps and factors involved. Despite this, the precise details of SL *trans*-splicing have yet to be elucidated. In part, this is because the systematic identification of the molecules involved has not previously been possible due to the lack of a specific phenotype associated with defects in this process. We present here a novel GFP-based reporter assay that can monitor SL1 *trans*-splicing in living *C. elegans*. Using this assay, we have identified mutants in *sna-1* that are defective in SL *trans*-splicing, and demonstrate that reducing function of SNA-1, SNA-2 and SUT-1, proteins that associate with SL1 RNA and related SmY RNAs, impairs SL *trans*-splicing. We further demonstrate that the Sm proteins and pICln, SMN and Gemin5, which are involved in small nuclear ribonucleoprotein assembly, have an important role in SL *trans*-splicing. Taken together these results provide the first *in vivo* evidence for proteins involved in SL *trans*-splicing, and indicate that continuous replacement of SL ribonucleoproteins consumed during *trans*-splicing reactions is essential for effective *trans*-splicing.

## INTRODUCTION

A wide range of eukaryotes engage in the *trans-*splicing of their pre-mRNAs, a process which results in the replacement of the 5′-end of the transcript with a short, ‘spliced leader’ (SL) (1,2). The essential mechanism of SL *trans-*splicing involves an intermolecular splicing event between the donor molecule, the SL RNA and the acceptor pre-mRNA, which possesses an intron-like 5′ untranslated region (5′ UTR), containing an unpaired 3′ splice site, termed the ‘outron’. However, most organisms that possess SL *trans-*splicing also contain a sub-set of genes organized into operons, consisting of several genes under the control of a shared promoter. Operons thus produce polycistronic transcripts, which are resolved into monocistronic mRNAs through SL *trans-*splicing coupled to 3′ end formation.

SL *trans-*splicing occurs on approximately 70% of mRNAs in *Caenorhabditis elegans*, including those that are derived from polycistronic transcripts (3). *C. elegans* uses two classes of SLs, SL1 and SL2, the former being *trans-*spliced to pre-mRNAs produced by monocistronic genes and to the pre-mRNAs derived from the first genes in operons (4). In contrast, SL2 is spliced exclusively to pre-mRNAs that are derived from downstream genes in operons (5). SL1 is essential, but lack of SL1 can at least in part be complemented by overexpression of SL2 RNA (6), presumably because they participate in fundamentally similar splicing events and the end products of these events are functionally similar. However, features that distinguish SL1 and SL2 *trans-*splicing have been defined, making it clear that these are distinct events (7–9).

SL RNAs form SL ribonucleoprotein particles (RNPs) and share some properties with the small nuclear RNPs (snRNPs) involved in *cis*-splicing reactions, in that they adopt similar secondary structures and are bound by proteins recognized by anti-Sm antibodies (10–13).

Additional proteins associated with SL RNAs were isolated from *Ascaris* splicing extracts, and homologs of these were identified in *C. elegans* (14,15). *C. elegans* SNA-2 was identified as the ortholog of the 95 kDa *Ascaris* protein, and was found to interact with SL1, but not SL2. The smaller, SL-30p *Ascaris* protein, was shown to have two *C. elegans* homologs, SNA-1 and SUT-1, with the former also interacting with SL1, while the latter was found to interact with a novel, nematode-specific class of snRNA termed SmY RNA (15,16). Subsequent work has shown that SNA-1 and SUT-1 are conserved throughout the Rhabditida (17). RNAi knockdown of *sna-1* or *sut-1* causes cold-sensitive sterility, and loss of both *sna-1* and *sut-1* function results in synthetic defects in viability, despite the fact that they show distinct biochemical properties (15).

To account for this MacMorris and colleagues have proposed that SNA-1 and SUT-1, together with SNA-2, associate with SL1 and SmY RNP complexes, respectively, and may function to recycle Sm proteins following *trans-*splicing (15). This hypothesis is based on the fact that SL *trans-*splicing consumes SL RNPs by transfer of the 5′ terminal spliced leader sequence onto mRNAs. According to this model, SNA-1 is involved in the recycling of Sm proteins following SL1 *trans-*splicing and thus loss of SNA-1 is compensated for by the presence of the other SUT-1/SmY-dependent Sm protein recycling pathway. Similarly, loss of SUT-1 would be tolerated because of the presence of the SNA-1/SL1 Sm recycling pathway. Loss of both SNA-1 and SUT-1 would compromise both pathways and thus results in a lethal loss of Sm protein recycling. Consistent with this model, loss of SNA-2, an essential component of both complexes, is lethal (15).

While this model explains the available molecular and genetic evidence, these novel proteins have not been directly shown to be required for SL *trans*-splicing *in vivo*. To better characterize SL *trans-*splicing we have designed a novel GFP reporter-based assay allowing us to genetically identify factors involved in this process in living *C. elegans*. We show that depletion of *sna-1, sna-2* and *sut-1* impairs SL *trans-*splicing *in vivo* and have identified new loss-of-function alleles of *sna-1* from a genetic screen using our assay. We also show that depletion of Sm protein expression, and factors involved in snRNP assembly, leads to reduced SL *trans*-splicing. A role for these factors in SL *trans*-splicing was confirmed by analyzing SL *trans*-splicing of the GFP reporter gene transcripts, and of an endogenous *trans*-spliced RNA, by qPCR. To our knowledge, our molecular analysis demonstrates for the first time in a living system a role for these factors in SL *trans*-splicing. SL RNPs consumed during *trans*-splicing need to be replaced and our observations imply that the synthesis of new Sm proteins makes a significant and crucial contribution to the pool of Sm proteins available for the assembly of new SL RNPs.

## MATERIALS AND METHODS

### Strains

Nematodes were maintained using standard protocols at 20°C on *Escherichia coli* B strain OP50 unless otherwise stated (18,19). The following strains were used: N2 (Bristol); PE720 [fex308]; PE774 [*sna-1(fe45)* V; *feEx308*]; PE766 [*sna-1(fe47)* V; *feEx308*]; PE793 [*feIs11* X]; FX3079 [*sut-1(tm3079*) II]. Transgenic genotypes are as follows: *feEx308* is an extrachromosomal array consisting of [*P_vit-2_*::*outron*::*gfp*^M1A^*P_myo-3_::*mCherry] (‘gfp^M1A^’ indicates that the initiator methionine of GFP has been changed to an alanine codon); *feIs11* was generated by γ-ray-mediated integration of the *feEx308* array into the X chromosome.

### Construction of SL *trans*-splicing reporter transgene and generation of transgenic strains

We amplified the *vit-2* promoter (P*_vit-2_*) from wild type (wt) genomic DNA and inserted the resulting amplicon into BamHI-XbaI cut pPD95.75 (gift from A. Fire, Stanford School of Medicine; available from Addgene; plasmid #1494) to give pAWF29B. A synthetic ‘OU141’ outron based on previous studies (20) was assembled from oligonucleotides with overlaps filled in by polymerase chain reaction (PCR), and inserted into pGEM-T Easy (Promega). The resultant plasmid was digested with AgeI and BamHI and the OU145 outron insert was cloned into AgeI-BamHI cut pAWF29B to produce pAWF30. The GFP initiation codon was changed to an alanine (GCT) by site directed mutagenesis using the Q5 Site-Directed Mutagenesis Kit (New England Biolabs), generating pLP6. The plasmid was sequenced to ensure no additional mutations were induced during the various cloning steps. This revealed that the OU141 outron sequence, which contained four copies of a 34 bp synthetic sequence (20) had undergone a truncation in pLP6 leaving only three copies (Supplementary Figure S2).

pLP6 was co-injected with pCFJ104, which expresses mCherry in body wall muscles (21). Five transgenic lines were generated, and all behaved similarly. One line, containing the extrachromosomal array *feEx308*, was selected for further study. The extrachromosomal array was integrated by subjecting transgenic animals to 3800 rad γ irradiation for 20 min (22). Four independent strains (*feIs11-14*) were recovered and backcrossed to wt six times. *feIs11* was mapped to the X chromosome and chosen for use in this study.

### Spliced leader *trans*-splicing reporter construct genetic screen

A population of PE720 worms were subject to ethyl methanesulfonate (EMS) mutagenesis (23) and allowed to become gravid adults. F1 progeny were picked singly to separate plates and their progeny screened using a stereomicroscope equipped with an epifluorescent light source for the presence of intestinal GFP expression. Individual fluorescent animals were then picked to fresh plates and their progeny scored to confirm the intestinal GFP expression phenotype.

Each mutant line was outcrossed with wt males and non-transgenic cross-progeny males crossed with unmutagenized PE720 hermaphrodites to ensure that the phenotype was not associated with changes to the transgenic array, and to determine the inheritance pattern of the mutation. Mutations in *sna-1* were identified by PCR amplification of exons followed by Sanger sequencing (DNA Sequencing and Services, University of Dundee).

### Epifluorescence microscopy

To determine the proportion of animals expressing GFP, ∼50 eggs were seeded on 3 cm RNAi plates with *E. coli* K-12 derived HT115(DE3) transformed with the appropriate feeding plasmid and allowed to grow at 20°C for 3 days to L4/adult stage. The total number of hatched animals and the number of GFP fluorescent animals were determined using a Leica MZ16F fluorescent stereomicroscope, and used to calculate the proportion of GFP expressing animals. To measure the intensity of GFP fluorescence, 10 animals were immobilized on slides with 1% agarose cushions, using 0.5% l-phenoxy-2-propanol as an anaesthetic, covered with a cover slip and sealed with Vaseline. A Zeiss Axioplan 2 fluorescence microscope was used to capture GFP and mCherry fluorescence for each animal. ImageJ software (24) was used to analyze the intensity of GFP and mCherry fluorescence. The GFP fluorescence intensity of each animal was normalized with respect to its mCherry fluorescence intensity. Statistical analysis by One-way ANOVA on ranks followed by Dunn's post-test was done using GraphPad Prism version 5.00 for Windows, GraphPad Software, San Diego, CA, USA, www.graphpad.com.

### RNA Interference

To prepare the RNAi feeding vectors, DNA was PCR amplified from genomic DNA or cDNA and inserted by In-Fusion cloning (Takara Bio Inc.) into the plasmid pPD129.36 cut with SpeI. The targeted genes and the primers used for PCR amplification are shown in the Supplementary Table S1. The mixtures were directly used to transform *E. coli* HT115. DNA sequences were verified by Sanger sequencing (DNA Sequencing and Services, University of Dundee). The *unc-22* RNAi construct was described earlier (25). RNAi-by-feeding was carried out as previously described (26), by seeding eggs, isolated by alkali hypochlorite treatment of gravid adults, onto RNAi plates. RNA was then prepared from L4/young adult animals.

### Reverse transcription-quantitative PCR (RT-qPCR)

Total RNA was prepared from *C. elegans* strains grown *on E. coli* using the PureLink RNA Mini kit (Life Technologies) with modifications for TRIzol treated samples and DNAse treatment as described by the manufacturer. For cDNA synthesis, total RNA was reverse transcribed using oligo(dT) Primers and M-MLV Reverse Transcriptase, according to the manufacturer's instructions. qPCR assays were designed using the Universal Probe Library Assay Design Centre (Roche) and tested for efficiency (Supplementary Table S2). qPCR was done in three technical replicates on a Roche Lightcycler 480 using standard settings. The Minimum Information for Quantitative Real-Time PCR Experiments (MIQE) is included as Supplementary Data. Data was analyzed using the comparative C_T_ method, assuming a primer efficiency of 2 (27). Outron levels were standardized with respect to internal RNA. Outron levels detected in *unc-22(RNAi)* treated animals were defined as 1.

## RESULTS AND DISCUSSION

### An *in vivo* assay to detect loss of SL1 *trans-*splicing

**Figure 1.**
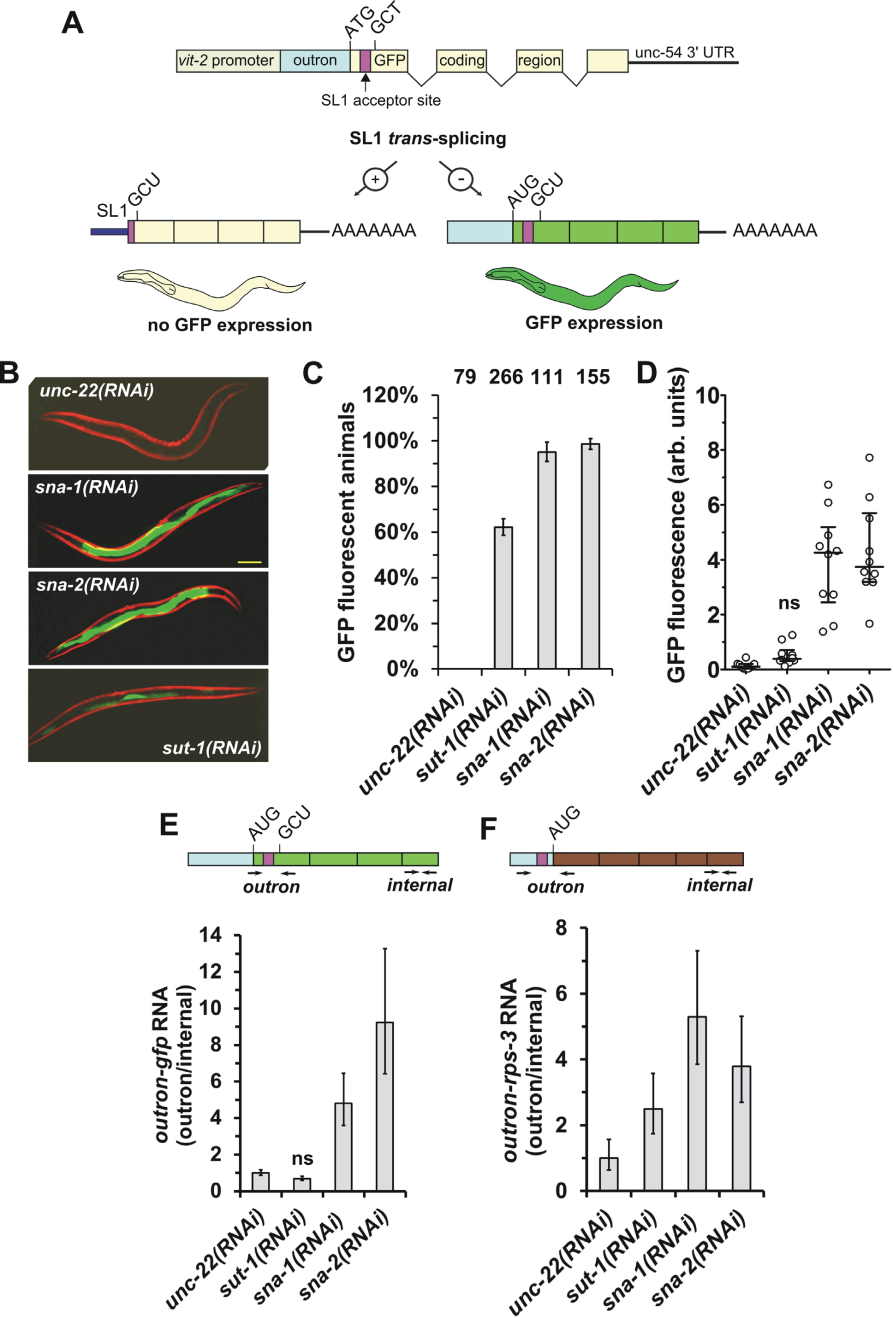
A novel reporter gene assay for the *in vivo* detection of reduced SL1 *trans-*splicing. (**A**) The *P_vit-2_*::*outron*::*gfp^M1A^* transgene engineered to monitor SL1 *trans*-splicing. The original ATG of the GFP open reading frame (yellow shading) was changed to a GCT alanine codon. Note the *gfp* gene contains three introns. The two mRNA products produced in the presence, or absence of SL1 *trans-*splicing are shown below, and a cartoon of the predicted GFP expression in wild-type (wt) worms (green shading indicates intestinal GFP fluorescence). (**B**) Detection of SL1 *trans*-splicing inhibition by fluorescence microscopy. Micrographs of GFP expression in animals carrying the *P_vit-2_*::outron::GFP^M1A^ transgene subjected to *sna-1, sna-2, sut-1* or *unc-22(RNAi)*. Exposure times were identical for all micrographs (5 ms). The scale bar corresponds to 100 μm. (**C** and **D**) Quantitation of GFP expression in animals carrying the *P_vit-2_*::outron::GFP^M1A^ transgene subjected to *sna-1, sna-2, sut-1* or *unc-22(RNAi)*. (C) The proportion of GFP fluorescent animals was determined by fluorescence microscopy. Error bars represent the standard deviation based on between two and five independent experiments (*unc-22(RNAi):* 2; *sna-1(RNAi), sna-2(RNAi)*: 3*; sut-1(RNAi)*: 5). The total number of animals analyzed is indicated at the top of the graph. (D) The intensity of GFP fluorescence (in arbitrary units) was determined from micrographs as shown in (B), and standardized with respect to mCherry fluorescence. The graph plots the GFP fluorescence of 10 animals analyzed for each RNAi treatment; shown are the median and the first and third quartile. ‘ns’ indicates values not significantly higher than in *unc-22(RNAi)* animals *(P* ≥ 0.05; ANOVA). (**E** and **F**) Detection of SL1 *trans*-splicing inhibition by RT-qPCR. Animals carrying the *P_vit-2_*::outron::GFP^M1A^ transgene were subjected to *sna-1, sna-2, sut-1* or *unc-22(RNAi)*, and *trans*-splicing of *gfp* reporter gene and *rps-3* transcripts was analyzed by reverse transcription followed by qPCR. The schematic diagrams indicate the position of the primers used (drawings are not to scale). Outron sequences are shown in blue and the SL1 acceptor sites in purple. Outron-*gfp* and outron-*rps-3* RNA levels were standardized with respect to an internal part of the mRNA (internal) and levels in *unc-22(RNAi)* animals were defined as 1. Note that elevated outron-*gfp* or outron-*rps-3* levels indicate inhibition of SL *trans*-splicing. ‘ns’ indicates values not significantly higher than in *unc-22(RNAi)* animals (*P* ≥ 0.05, *t-*test). Error bars show standard deviation from three technical replicates. Similar results were obtained in two independent experiments.

We generated a transgenic strain that would display GFP fluorescence dependent on suppression of SL *trans-*splicing (Figure 1A). The transgene consists of a modified GFP gene that is under the transcriptional control of the *vit-2* promoter (20). The GFP gene was modified such that the original ATG initiation codon was changed into an alanine codon, GCT (GFP^M1A^). Upstream of this we engineered a synthetic outron sequence, which has previously been shown to confer on a pre-mRNA the ability to act as SL1 *trans-*splicing substrate (20). We placed a new ATG codon in-frame with the GFP coding region upstream of the 3′ splice site. Thus, in wild type worms, the removal of the synthetic outron from the pre-mRNA during SL *trans*-splicing would be expected to produce an mRNA lacking an AUG, and so no GFP fluorescence would be observed, whereas in animals with reduced SL *trans-*splicing, mRNA retaining the outron and its associated in-frame AUG would be predicted to accumulate, and thus allow GFP expression (Figure 1A). As predicted, transgenic lines established with this P*_vit-2_*::outron::GFP^M1A^ construct displayed only sporadic, weak fluorescence (Figure 1B–D: *unc-22(RNAi)*).

To test the response of the transgenic strain to loss of SL *trans-*splicing, we carried out RNA interference (RNAi) targeted against *sna-1* and *sna-2*, since these genes encode known components of the SL RNP, and *sut-1*, because of its genetic interaction with *sna-1* and proposed role in Sm protein recycling (15). We observed a strong GFP expression response in animals subjected to *sna-1(RNAi)* and *sna-2*(*RNAi)*, indicating that our assay is responding as expected (Figure 1B–D). We observed GFP expression in more than 60% of *sut-1(RNAi)* treated animals. While fluorescence was clearly detectable, it was weak and not significantly above the background values obtained in *unc-22(RNAi)* controls (Figure 1B–D), largely because typically relatively few intestinal cells displayed fluorescence in *sut-1(RNAi)* animals. To confirm that GFP expression correlates with retention of the outron in the GFP mRNA we used qRT-PCR to measure the levels of non-*trans*-spliced outron sequences, and of an internal part of the reporter gene transcript not involved in SL *trans*-splicing. Relative *trans*-splicing efficiencies were calculated by standardizing the levels of outron containing transcripts with respect to the internal fragments, which allows a direct comparison between different RNAi treatments (Figure 1E). This was complemented by the analysis of the SL *trans*-splicing of endogenous *rps-3* transcripts, which are known to be *trans*-spliced by SL1 (3) (Figure 1F). In animals treated with *sna-1(RNAi)* and *sna-2(RNAi)*, the levels of non-*trans*-spliced GFP reporter transcripts (from here on referred to as outron-*gfp* RNA) were significantly higher than in the *unc-22(RNAi)* treated control animals, reflecting the increase in GFP expression observed (Figure 1B–D). Furthermore, the levels of non-*trans*-spliced *rps-3* RNA (from here on referred to as outron-*rps-3* RNA) were elevated in *sna-1* and *sna-2 (RNAi)* treated animals (Figure 1F), indicating that the knockdown of these genes also interferes with the *trans*-splicing of endogenous transcripts. In animals subjected to *sut-1(RNAi)* we were not able to detect a significant increase of outron-*gfp* transcripts, reflecting the weak activation of the reporter observed by microscopy (Figure 1B–D). However, the level of outron-*rps-3* transcripts was elevated, consistent with a role of *sut-1* in SL *trans*-splicing.

**Figure 2.**
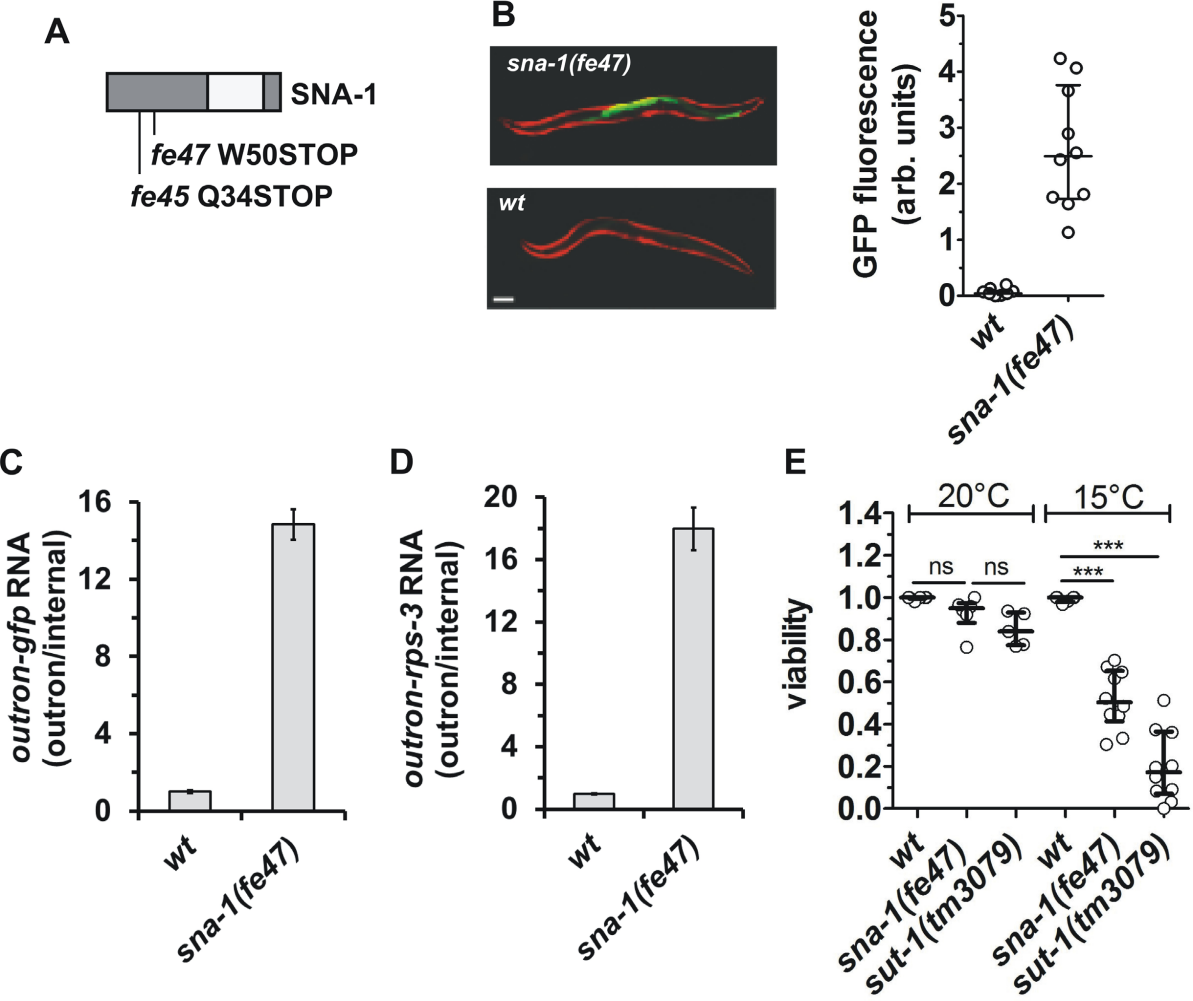
Loss-of-function mutations in *sna-1* recovered from a mutagenesis screen for genes involved in SL1 *trans*-splicing. (**A**) Schematic representations of the location of the residues affected by the mutations in the SNA-1 protein. The regions in dark gray are conserved in nematode SNA-1 orthologues (Supplementary Figure S1). (**B**) Representative images of wild type (wt) and *sna-1(fe47)* animals transgenic for *P_vit-2_*::*outron*::*gfp^M1A^*, showing intestinal GFP (green) expression and constitutive mCherry (red) expression in body wall muscle cells. Scale bar represents 100 μm. The graph plots the GFP fluorescence of 10 animals analyzed; shown are the median and the first and third quartile. (**C** and **D**) RT-qPCR analysis of SL1 *trans-*splicing in wt and *sna-1(fe47)* animals carrying the *P_vit-2_*::outron::gfp^M1A^ transgene. *Trans*-splicing of *gfp* reporter gene (C) and *rps-3* transcripts (D) was analyzed as described in the legend of Figure 1, with levels in wt animals set as 1. Error bars indicate standard deviation from three technical replicates. (**E**) Viability of wt, *sna-1(fe47)* and *sut-1(tm3079)* animals at 20 and 15°C, as expressed by the proportion of eggs that develop to L4/adult stage in 5–10 independent experiments. (***P ≤ 0.001; ‘ns’ not significant; ANOVA). Note that while at 20°C there is a slight reduction in viability in *sut-1(tm3079)* animals, this is not significantly different from the viability of *sna-1(fe47)* animals.

As further support for the role of SNA-1 in SL *trans*-splicing, we identified two loss-of-function *sna-1* mutations from a genetic screen for PE720 worms that displayed elevated levels of GFP fluorescence. The *fe45* allele is a C to T transition on chromosome V at position 6,710,253, creating a premature stop at codon 34 (CAA > TAA, Q34STOP) and *fe47* is a C to T transition on chromosome V at position 6,710,203, creating a premature stop codon at codon 50 (GGA > TGA; W50STOP) (Figure 2). Detailed analysis of GFP fluorescence was carried out for *sna-1(fe47)* homozygotes (Figure 2B). In addition, the levels of outron-*gfp* and outron-*rps-3* transcripts were more than 15-fold higher than in wild type animals (Figure 2C and D), confirming the inhibition of SL *trans*-splicing in these animals.

Although the two *sna-1* alleles that we isolated are viable, previous studies have shown that *sna-1(RNAi)* results in cold-sensitive sterility defects and that combined loss of *sna-1* and *sut-1* function results in embryonic lethality (15). We replicated these observations with the *sna-1(fe47)* allele, demonstrating that it confers cold-sensitive defects in viability, similar to the *sut-1(tm3079)* allele (Figure 2E). Furthermore, *sut-1; sna-1* double mutants are sickly, with the majority (92.5%; *n* = 40) being completely sterile.

In conclusion, these experiments clearly demonstrate that reduced expression of *sna-1* or *sna-2* interferes with SL *trans*-splicing. The genetic interaction of *sna-1* with *sut-1*, combined with the weak disturbance of SL *trans*-splicing by *sut-1* depletion supports a function of *sut-1* in SL *trans*-splicing. It has been demonstrated that SUT-1 interacts with SmY RNAs (15), and this provides further support for a functional role of SmY RNAs in SL *trans*-splicing. Taken together, our data demonstrate a role for these molecules in SL *trans*-splicing, as proposed in earlier molecular studies.

Previous biochemical analyses of SL RNPs using anti-Sm antisera imply that SL1 RNA is associated with Sm proteins (10,13,15). However, since the post-translational modification of Sm proteins, symmetrical dimethylation of arginines, is the major epitope for anti-Sm antibodies (28), it is possible that other proteins containing this epitope, such as Lsm4 or piRNA associated Mili protein, might explain the ability of anti-Sm antisera to interact with SL RNPs (29,30). It was therefore important to directly test the role of Sm proteins in SL *trans*-splicing.

Sm proteins form heptameric rings that bind to a variety of snRNAs and are found in snRNPs acting in, for example, mRNA splicing. The major U1, U2, U4 and U5 snRNAs associate with a ring formed by the Sm proteins B, D1, D2, D3, F, E and G. In *C. elegans*, these proteins are encoded by *snr-1, snr-2, snr-3, snr-4, snr-5, snr-6* and *snr-7*, respectively (31). In addition, the related LSm proteins form heptameric rings that interact with other RNAs (32). U6 snRNA, involved in *cis-*splicing is bound by a ring formed from LSm2, 3, 4, 5, 6, 7, 8 proteins, which in *C. elegans* are encoded by the *gut-2, lsm-3, lsm-4, lsm-5, lsm-6, lsm-7* and *lsm-8* genes, respectively. A similar ring where mammalian LSm8 is replaced by LSm1 (encoded by *lsm-1*) is involved in the degradation of mRNA (32). Using similarity searches we also identified the genes *K07A1.15, C49H3.4* and *M142.5* as encoding proteins with Sm-like domains (33). *M142.5* was identified by others as an orthologue of human LSm12 (34), while *K07A1.15* is annotated in Wormbase as the orthologue of human LSm10. LSm10 protein associates with LSm11 and Sm proteins B, D3, G, E and F to form the U7 snRNP particle involved in histone RNA processing (35,36). *Drosophila* Lsm10 and Lsm11 mutants die as pupae (37), indicating that these functions are essential. Surprisingly, no phenotype has been attributed to *K07A1.15* loss-of-function (33,38,39). We thus wanted to determine the effect of knocking down the expression of Sm and LSm protein function on GFP fluorescence in our reporter strain, as well as on the levels of outron-*gfp* and outron-*rps-3* transcripts.

**Figure 3.**
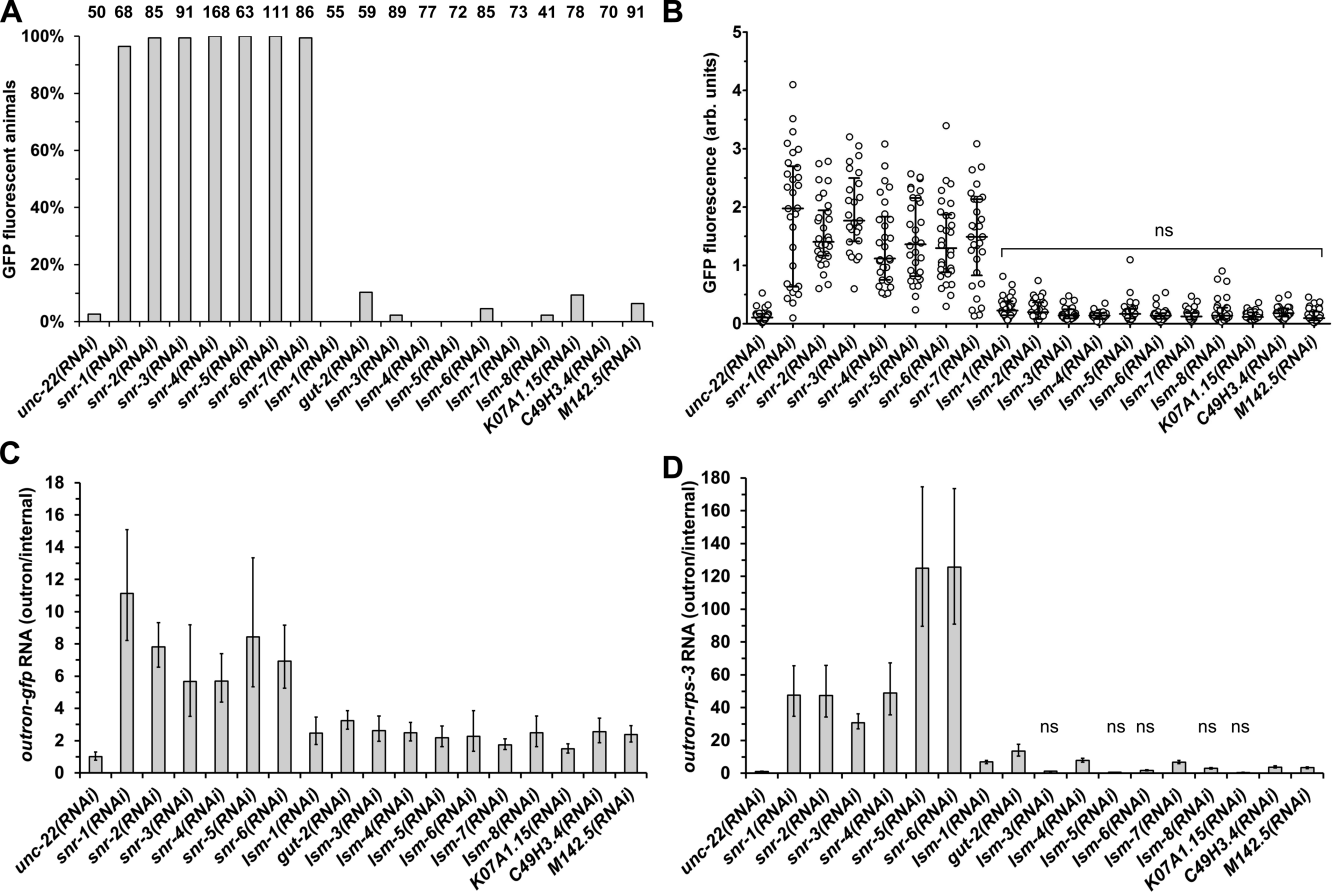
SL1 spliced leader *trans*-splicing is sensitive to the knockdown of *snr*, but not *lsm*, gene transcripts. Detection of SL1 *trans*-splicing inhibition by fluorescence microscopy. Quantitation of GFP expression in animals carrying the *P_vit-2_*::outron::GFP^M1A^ transgene subjected to RNAi was done as described for Figure 1. (**A**) The proportion of GFP fluorescent animals. The number of animals analyzed are indicated above each dataset. (**B**) GFP fluorescence intensity. The graph summarizes individual measurements (*n* = 30) from three independent experiments. The distribution of measurements for each of the experiments is similar to the distribution of the pooled data. Median and the first and third quartile are indicated. ‘ns’ indicates values not significantly higher than in *unc-22(RNAi)* animals (*P* ≥ 0.05; ANOVA). (**C** and **D**) Detection of SL1 *trans*-splicing inhibition by RT-qPCR. Animals carrying the *P_vit-2_*::outron::GFP^M1A^ transgene were subjected to RNAi, and *trans*-splicing of the *gfp* reporter gene (C) and *rps-3* transcripts (D) was analyzed by reverse transcription followed by qPCR. Outron-*gfp* and outron-*rps-3* transcript levels were standardized as described in the Figure 1 legend, and levels in *unc-22(RNAi)* animals were defined as 1. Note that *snr-7(RNAi)* was not analyzed by RT-qPCR. ‘ns’ indicates values not significantly higher than in *unc-22(RNAi)* animals (*P* ≥ 0.05; *t-*test). Error bars show standard deviation from three technical replicates.

Figure 3 shows that the knockdown of *snr* gene transcripts led to GFP fluorescence in all, or nearly all animals tested, with a more than seven-fold increase of fluorescence compared to the *unc-22(RNAi)* treated control animals (Figure 3A and B). This was accompanied by five-fold or higher increase of outron-*gfp* transcripts in animals subjected to *snr(RNAi)* (Figure 3C). Importantly, *snr(RNAi)* also impaired *trans*-splicing of *rps-3* transcripts (Figure 3D). The effect on these transcripts appeared more severe than the effect on the reporter gene transcripts, with up to more than 100-fold higher levels of non-*trans*-spliced transcripts detected in *snr-5* and *snr-6(RNAi)* treated animals compared to the control treatment.

In contrast to the knockdown of Sm protein transcripts, knockdown of each of the eleven LSm protein transcripts failed to cause notable GFP fluorescence (Figure 3A and B). A low proportion of animals, not exceeding 10%, expressed GFP at a detectable level. This is distinct from the effect of *sna-1* and *sna-2(RNAi)*, and the knockdown of *snr* RNAs, which results in clearly detectable GFP fluorescence in all or most animals subjected to the RNAi treatments (Figures 1C and D, 3A and B). It is also distinct from *sut-1(RNAi)*, which resulted in 62% of animals expressing GFP, albeit at a low level (Figure 1C and D). In addition, the levels of non-*trans*-spliced transcripts in these animals were generally low. Outron-*gfp* transcript levels varied between 1.5- and 3.3-fold of levels observed in the *unc-22(RNAi)* control treated animals, while levels of outron-*rps-3* transcripts varied between 0.5- and 13.6-fold of those observed in the control treated animals, and were in many cases not significantly different from the control (Figure 3D).

The analysis of GFP fluorescence and that of *trans*-splicing by qRT-PCR were done independently. The difference between fluorescence and the detection of non-*trans*-spliced transcripts by qPCR in, for example, many of the *lsm(RNAi)* treatments may be caused by different sensitivities of the two methods. Importantly, the combination of the two approaches, with the inclusion of the analysis of *trans*-splicing of an additional endogenous gene, provides a coherent picture of the effect of the treatments.

Our findings indicate that SL1 *trans*-splicing is highly sensitive to the knockdown of the genes encoding Sm proteins: the knockdown of *snr* genes, similar to the knockdown of *sna-1* and *sna-2*, results in strong GFP fluorescence in the majority or all animals subjected to the RNAi treatments. This is supported by the significant increase of non-processed outron-*gfp* and outron-*rps3* RNA in these animals, which also indicates that *trans*-splicing is impaired. In contrast, *lsm* gene knockdown only results in weak GFP fluorescence in a small number of the treated animals. The molecular analysis of *trans*-splicing confirms that any GFP fluorescence detected is due to a defect in *trans*-splicing. Together these data indicate that SL *trans*-splicing is highly sensitive to the knockdown of the genes encoding Sm proteins, while the knockdown of genes encoding LSm proteins has no consistent detectable effect.

Since the Sm proteins are also required for assembly of *cis*-splicing U snRNPs, one possible interpretation is that the loss of these factors, all of which apart from U1 are required for SL *trans*-splicing (40,41), could contribute to the impaired SL *trans*-splicing that we observe. However, two observations suggest that the depletion of Sm proteins primarily impacts upon SL RNP function in our experiments. First, the removal, by *cis*-splicing, of the three GFP transgene introns, two of which contain stop codons, is required for fluorescence, implying that *cis*-splicing factors are not significantly impaired by Sm protein depletion. Second, depletion of multiple LSm proteins, which are required for U6 snRNP formation, has minimal effects on SL *trans*-splicing, despite the fact that U6 is required for *trans*-splicing (42). Thus, the most likely explanation for the impaired SL *trans*-splicing in animals depleted for Sm proteins is due to the effect of this loss on the formation of the SL RNP. This raises the question: why would *cis*-splicing U snRNPs be less sensitive to Sm protein depletion than SL RNPs? One explanation may be that the latter are consumed by the splicing reaction in which they participate, and thus SL *trans*-splicing is dependent on the continuous formation of new SL RNPs. As described earlier, it has been proposed that Sm protein recycling contributes to the formation of new SL RNPs (15). Our findings indicate that this is not sufficient, and that the formation of new SL RNPs is heavily reliant on the synthesis of new Sm proteins. An alternative, but not mutually exclusive, explanation is that SL RNA is stabilized by the integration into RNPs and is therefore sensitive to a reduction in RNP components.

Recycling of the snRNPs after completion of *cis*-splicing is an important, but poorly understood process. Investigations have mainly focused on the U6 snRNP, which undergoes major changes during the *cis*-splicing reaction and is associated with LSm protein (43,44). Our findings are compatible with a model where U6 snRNA and associated LSm proteins participating in SL *trans*-splicing are recycled, and do not require continuous replacement.

To determine whether factors involved in snRNP assembly also play a role in SL *trans*-splicing, we knocked down the expression of *smn-1, smi-1, icln-1* by RNAi. *smn-1* encodes a homolog of human SMN and *smi-1* an ortholog of human Gemin2 that interacts with SMN, and, together with SMN, is a component of the SMN complex (45). This complex mediates the specific assembly of the heptameric Sm protein ring onto snRNAs and interacts with both Sm proteins and snRNAs (46). *icln-1* encodes a homolog of the human methylosome subunit, pICln. Sm proteins, B/B’, D1 and D3 are modified by symmetrical dimethylation of arginine residues by a complex containing pICln, which promotes the interaction with the SMN complex for snRNP assembly (29,47,48).

**Figure 4.**
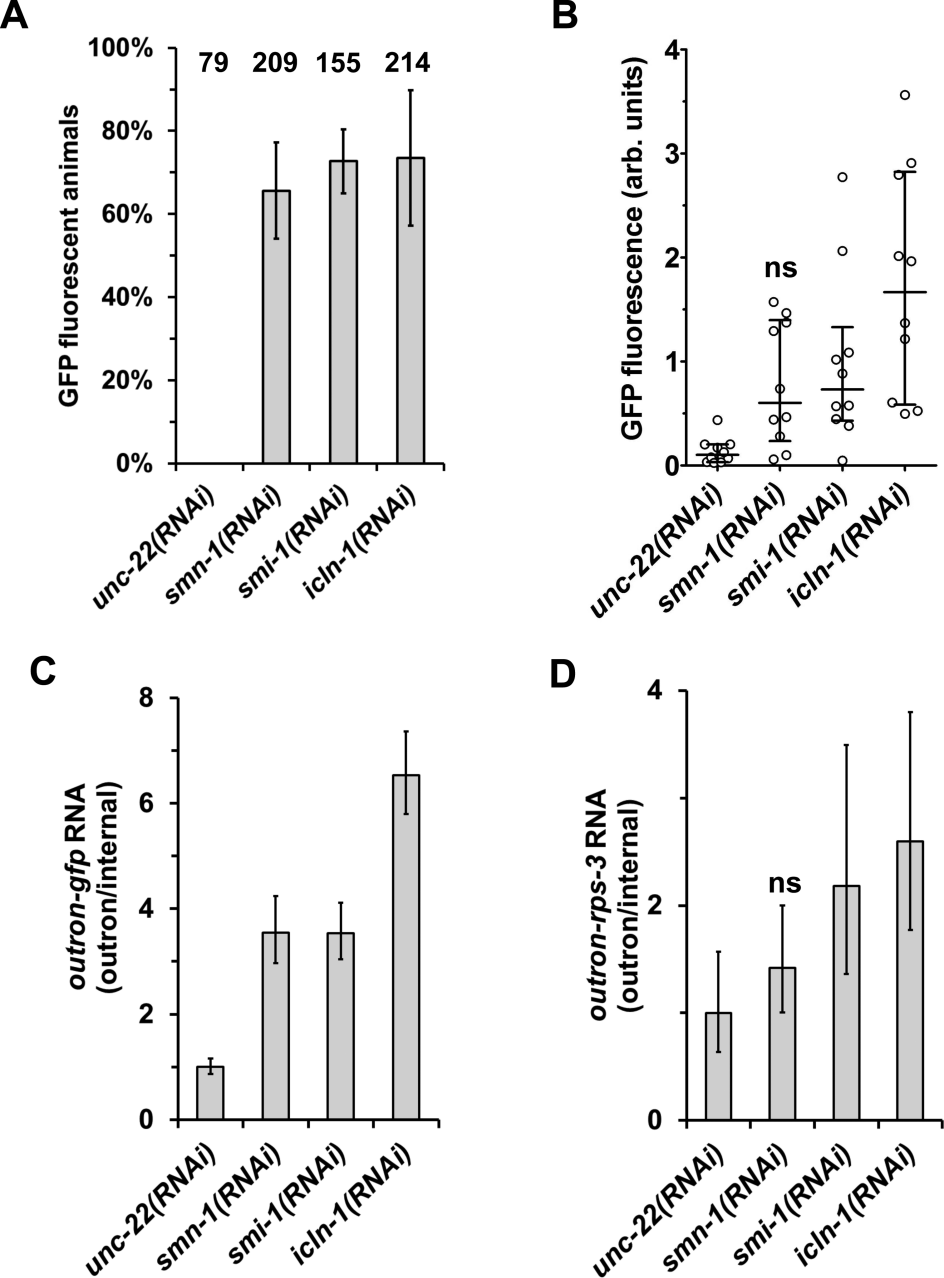
Knockdown of snRNP assembly factors inhibits SL1 spliced leader *trans*-splicing. Detection of SL1 *trans*-splicing inhibition by fluorescence microscopy. Quantitation of GFP expression in animals with the *P_vit-2_*::outron::GFP^M1A^ transgene subjected to RNAi was done as described for Figure 1. (**A**) The proportion of GFP fluorescent animals. The graph summarizes results from two or five independent experiments (*unc-22(RNAi):* 2; *smn-1(RNAi), smi-1(RNAi), icln-1(RNAi)*: 5). The total number of animals analyzed is indicated. (**B**) GFP fluorescence intensity. The graph shows measurements of fluorescence intensity of 10 animals analyzed for each RNAi treatment. ns indicates values not significantly higher than in *unc-22(RNAi)* animals (*P* ≥ 0.05; ANOVA). (**C** and **D**) Detection of SL1 *trans*-splicing inhibition by RT-qPCR. Animals carrying the *P_vit-2_*::outron::GFP^M1A^ transgene were subjected to RNAi, and *trans*-splicing of the *gfp* reporter gene (C) and *rps-3* transcripts (D) was analyzed by reverse transcription followed by qPCR. Outron-*gfp* and outron-*rps-3* transcript levels were standardized as described in the Figure 1 legend, and levels in *unc-22(RNAi)* animals were defined as 1. ‘ns’ indicates values not significantly higher than in *unc-22(RNAi)* animals (*P* ≥ 0.05, *t-*test). Error bars show standard deviation from three technical replicates.

Knockdown of *smn-1, smi-1* and *icln-1* led to GFP fluorescence in most of the animals subjected to RNAi, similar to the knockdown of *sna-1, sna-2* and the *snr* genes described above (Figures 1C and D, 3A and B), with *icln-1(RNAi)* giving the strongest effect (Figure 4A and B). The inhibition of SL *trans*-splicing by *smi-1* and *icln-1* knockdown was confirmed by the increased levels of outron-*gfp* and outron-*rps-3* transcripts (Figure 4C and D), indicating that the knockdown of these genes inhibits SL *trans*-splicing. *smn-1* knockdown caused a weaker effect: while reporter gene activation was detected in the majority of animals, the fluorescence was not strong enough to produce a significant difference in the quantitative comparison to *unc22(RNAi)* animals (Figure 4A and B). However, the increase of non-processed outron-*gfp* RNA in *smn-1(RNAi)* animals indicates that *trans*-splicing is impaired. These data suggest that the same biogenesis factors involved in snRNP assembly are required for these processes during the lifecycle of SL RNPs, further reinforcing the similarities between the U snRNPs and SL RNPs.

In conclusion, we have developed an *in vivo* assay that allows us to monitor the levels of SL *trans*-splicing in *C. elegans*, and have confirmed that there is a strong correlation between GFP fluorescence and outron-retention in SL *trans*-spliced mRNAs. This has allowed us to demonstrate physiological roles in SL *trans*-splicing for SNA-1, SNA-2 and SUT-1, and for Sm proteins and snRNP assembly factors. Our findings indicate that Sm protein recycling is not sufficient to replace the SL RNPs used up during *trans*-splicing, but that the assembly of new SL RNPs is heavily reliant on the synthesis of new Sm proteins. Furthermore, we have shown that this assay system can be employed to identify mutations in genes specifically involved in SL *trans*-splicing. Further application of such screens, including searches for lethal mutations, should allow the comprehensive identification of molecules involved in this fundamental nematode process. Finally, we envisage that this will be useful as a bioassay in the search for compounds that inhibit SL *trans*-splicing.

## Supplementary Material

Supplementary Data
