# Supplementary material for: An in vivo genetic screen for genes involved in spliced leader trans-splicing indicates a crucial role for continuous de novo spliced leader RNP assembly
Source: Nucleic Acids Res. 2017 Jun 5;45(14):8474–83. doi: 10.1093/nar/gkx500 (PMC5737717; doi:10.1093/nar/gkx500)
Supplement: Supplementary Data [file gkx500_supp.pdf]

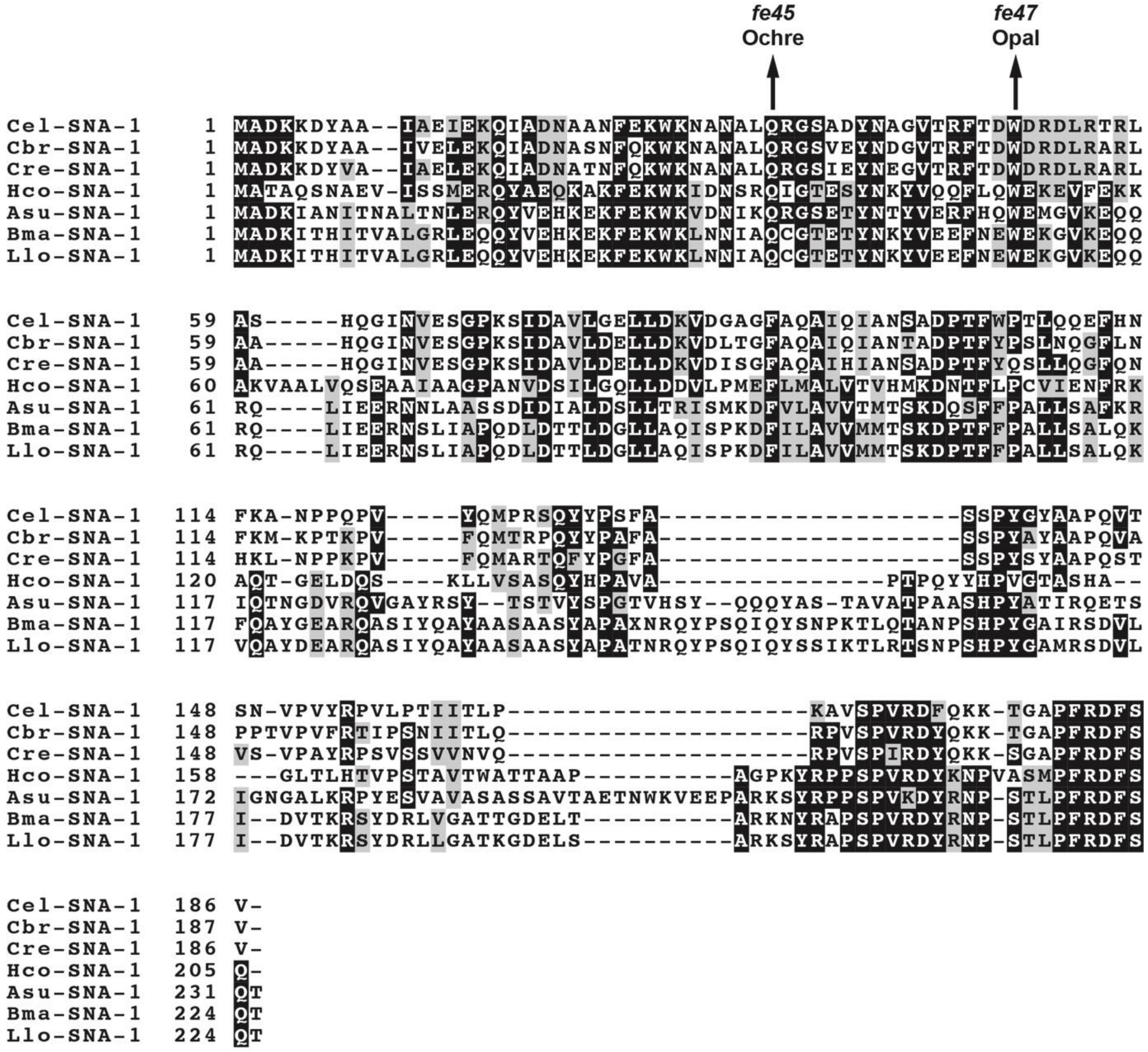

Supplementary Figure 1. Multiple sequence alignment of SNA-1 homologues. Arrows indicate the residues affected by the two *sna-1* mutations, and the nature of the resultant nonsense mutation. Residues conserved in more than 50% of the sequences are shaded (counter shading indicating identity and grey shading indicating similarity). Species key: Asu – *A. suum*; Bma – *B. malayi*; Cbr – *C. briggsae*; Cel – *C. elegans*; Cre – *C. remanei*; Hco – *H. contortus*; Llo – *L. loa*.

acggctcgattaaaattaaaattaaaattatcgaacggctcgattaaaattaaaattaaaat

tatcgaacggctcgattaaaattaaaattaaaattatc**ATG**ttttcaggaccggtagaaaaa

GCTagtaaaggagaagaacttttcactggagttgtcccaattcttgttgaattagatggtga

tgttaatgggcacaaattttctgtcagtggagagggtgaaggtgatgcaacatacggaaaac

ttacccttaaatttatttgcactactggaaaactacctgttccatgggtaagtttaaacata

tatatactaactaaccctgattatttaaattttcagccaacacttgtcactactttctgtta

tggtgttcaatgcttctcgagatacccagatcatatgaaacggcatgacttttcaagagtg

ccatgcccgaaggttatgtacaggaaagaactatatttttcaaagatgacgggaactacaag

acacgtaagtttaaacagttcggtactaactaaccatacatatttaaattttcaggtgctga

agtcaagtttgaaggtgataccctt gttaatagaatcgagttaaaaggtattgattttaaag

aagatggaaacattcttggacacaaattggaatacaactataactcacacaatgtatacatc

atggcagacaaacaaaagaatggaatcaaagttgtaagtttaaacatgattttactaactaa

ctaatctgatttaaattttcagaacttcaaaattagacacaacattgaagatggaagcgttc

aactagcagaccattatcaacaaaatactccaattggcgatggccctgtccttttaccagac

aaccattacctgtccacacaatctgccctttcgaaagatcccaacgaaaagagagaccacat

ggtccttcttgagtttgtaacagctgctgggattacacatggcatggatgaactatacaaat

ag

Supplementary Figure 2. Sequence of the *gfp* reporter gene with outron. The original *gfp* start codon converted to GCT is shown in capital letters. The new ATG initiation codon is shown in red capital letters and is followed by a *trans*-splice site (underlined). Intron sequences are printed in grey.   Arrows indicate positions of the primers for the detection of outron *gfp* mRNA (green) and of internal *gfp* mRNA sequence (blue).

Target	Primer 1	Primer 2
smn-1	CCGCTCTAGAACTAGTGCGACATGGAGGTAGACGAT	CGGTGGATCCACTAGTCGGGAGGAATGGAGTGAGAA
smi-1	CCGCTCTAGAACTAGTGATCAAGAAGCCTGTCTCGG	CGGTGGATCCACTAGTAAAACAACGCGGAGAAGGAG
icln-1	CCGCTCTAGAACTAGTTGAAGTCAGCCAGCCAACT	CGGTGGATCCACTAGTCACGGGTCAATAACAACATGGA
F23F1.5	CCGCTCTAGAACTAGTAAGCTGCAGAACAACAAGCA	CGGTGGATCCACTAGTACCCAAAACGGTCCAAAACT
snr-1	CCGCTCTAGAACTAGTTTCATGAGGCCGAAGGTCACA	CGGTGGATCCACTAGTATCTTACAGGGAAATCTCCGAAACC
snr-2	CCGCTCTAGAACTAGTCCAACGCCTCAAGATGACTATC	CGGTGGATCCACTAGTCGAAGATTGGTGGGTGATGG
snr-3	CCGCTCTAGAACTAGTTAGAAAAATGAAGTTGGTCAGATTCC	CGGTGGATCCACTAGTATTGAAGAAATTTATAGAGATCAGAACG
snr-4	CCGCTCTAGAACTAGTAGCCAAACCCCGTTCAGAGATG	CGGTGGATCCACTAGTCATTGAAACGAAACAACAGGGTGAAC
snr-5	CCGCTCTAGAACTAGTAATGTCCGCAGTTCAACCAG	CGGTGGATCCACTAGTGATAATTATAATTTATTGATTAAGAATGTGC
snr-6	CCGCTCTAGAACTAGTGAAAGCTCAACAAAGTGATGGTTCAG	CGGTGGATCCACTAGTTTAGGCTTCTTGTTGGGCGG
snr-7	CCGCTCTAGAACTAGTAAAATGAGTAAGACACATCCACCAG	CGGTGGATCCACTAGTGAGTTTCCGCGAATGACAG
lsm-1	CCGCTCTAGAACTAGTGCCCGATCCCTATTTACCC	CGGTGGATCCACTAGTATTCATGCATCGTGTTCTTCTG
gut-2	CCGCTCTAGAACTAGTATGCTGTTCTTCTCATTCTTCA	CGGTGGATCCACTAGTCGTGGATTTACTGTTTTGCC
lsm-3	CCGCTCTAGAACTAGTGGCCACCGAAAAGAAAGAAG	CGGTGGATCCACTAGTAATGATGACGATGAATTAGGATGC

lsm-4	CCGCTCTAGAACTAGTATGGTGTTGCCACTTTCTCTTCT	CGGTGGATCCACTAGTCGTCCTCCTCGTCCACCA
lsm-5	CCGCTCTAGAACTAGTCCATGGCAACCTCAACATC	CGGTGGATCCACTAGTTTAAATCTCTGGTCCTTCTCCTC
lsm-6	CCGCTCTAGAACTAGTGAAAATGAGCAAACGACAGAATC	CGGTGGATCCACTAGTTTATTTCGTCGATGTGGAGATGT
lsm-7	CCGCTCTAGAACTAGTGAAAGACGAAGGAAAACGAAAG	CGGTGGATCCACTAGTTTATTCTTCTTCCTGGGTTGC
lsm-8	CCGCTCTAGAACTAGTAATATGACTTCAACTCTAGATGCGTA	CGGTGGATCCACTAGTTGTGGAAATTATTGGGGAATC
K07A1.15	CCGCTCTAGAACTAGTGACTCGAATCTGGATGTGGTGC	CGGTGGATCCACTAGTCTGTTTCTGATCAAGTCGCCG
M142.5	CCGCTCTAGAACTAGTTTCGACACGACTCGCAAGGTT	CGGTGGATCCACTAGTGCCACAATAGCTCCAATAGTGAAATC
C48H3.4	CCGCTCTAGAACTAGTGAAGGGGTCGAAGGAAATAGTG	CGGTGGATCCACTAGTGAACAGTTTCCGTAACATCTTTCATC

Supplementary Table S1. Primers for the amplification of targets for RNAi feeding vector production. Sequences derived from pPD129.36 and included for In-Fusion cloning are underlined.

qPCR assays							
Target	Primers	Primer location[1]	Similarity to *E. coli* [A]	UPL probe (sequence)	Amplicon Length	Efficiency [B]	R^2
Outron *gfp* mRNA	TAAAATTATCATG TTTTCAGGAC	*gfp* reporter (supplementary Figure 2)	no primer binding sites	9 (TGGTGATG)	147 bp	1.982	0.957
	GTTGCATCACCTT CACCCTC	*gfp* reporter (supplementary Figure 2)	no primer binding sites				
Internal *gfp* mRNA	CCACATGGTCCTT CTTGAGTTT	*gfp* reporter (supplementary Figure 2)	no primer binding sites	3 (CTGCTGGG)	62 bp	1.969	0.955
	ATAGTTCATCCAT GCCATGTGTA	*gfp* reporter (supplementary Figure 2)	no primer binding sites				
Outron-*rps-3* mRNA	TATATTTCTTGTG TTTTGTTCGGATT	chrIII: 6206726-6206751	no primer binding sites	46 (ATGGCTGC)	140 bp	1.932	0.993
	ACGGCCTTCTTCT TCTTGGT	chrIII: 6206846-6206865	no primer binding sites				
Internal *rps-3* mRNA	TCCTTCCAAAGGA ACCACAC	chrIII: 6207842-6207861	no primer binding sites	63 (AGGAGGA)	62 bp	1.967	0.993
	TGCTGGGACTTGA ACATCCT	chrIII: 6207884-6207903	no primer binding sites				

Supplementary Table S2. qPCR assays were designed using the Universal Probe Library Assay Design Centre (Roche). [A] BLAST searches were done at NCBI against *Caenorhabditis elegans* (taxid:6239) , *Escherichia coli* K-12 (taxid:83333) and OP50 (taxid:637912). No *E. coli* sequences with ≥75% sequence identity were found, and primers, and primer combinations, were specific for *gfp* and *rps-3* mRNA. Each primer pair was tested on serial template dilutions. 10-fold serial dilutions ofLP6 plasmid (covering a range from 0.1 ng/µl to 1 fg/µl) were used for primers detecting the *gfp* reporter mRNA, and 10-fold serial dilutions of N2 genomic DNA (covering a range from 70 ng/µl to 70 fg/µl) for primers detecting *rps-3* mRNA. Cq values of the dilution series were plotted in Microsoft Excel and used for linear regression curve fitting. The qPCR primer Efficiency [(B)] was calculated from the resulting slope according to the equation: $E = (10^{[-1/slope]})$. R^2 calculated for the linear regression is also show.

PCR assays			
Target	Primers	Primer location	Amplicon Length (genomic DNA/cDNA)
act-1,<br>*act-2,*<br>*act-3*	CGTGGTTACTCTTTCACCACCACCGCT	chrV:11,081,709-11,081,735<br>chrV:11,077,468-11,077,494<br>chrV:11,072,581-11,072,607	*act-1*: 562/510 bp<br>*act-2*: 561/510 bp<br>*act-3*: 563/509 bp
	GGACTCGTCGTATTCTTGCTTGGAGAT	chrV:11,082,244-11,082,270<br>chrV:11,076,934-11,076,959<br>chrV:11,072,045-11,072,071	
dab-1	TATGGACGCATTGGTTGGT	chrII:8,228,308-8,228,326	228/100 bp
	CGACATGGAGTTCAGAGAAGC	chrII:8,228,515-8,228,535	

Supplementary Table S3. PCR assays. The PCR assay for the detection of actin transcripts has been described elsewhere (1).

REFERENCES

1. Keall,R., Whitelaw,S., Pettitt,J. and Müller,B. (2007) Histone gene expression and histone mRNA 3' end structure in *Caenorhabditis elegans. BMC Mol Biol.*, **8**, 51.
